# Shedding New Light on The Role of ανβ3 and α5β1 Integrins in Rheumatoid Arthritis

**DOI:** 10.3390/molecules24081537

**Published:** 2019-04-18

**Authors:** Arwa Morshed, Abdul Baset Abbas, Jialiang Hu, Hanmei Xu

**Affiliations:** 1 The Engineering Research Center of Synthetic Polypeptide Drug Discovery and Evaluation of Jiangsu Province, China Pharmaceutical University, Nanjing 210009, China; arwamohammed111@hotmail.com (A.M.); abduabbas204@hotmail.com (A.B.A.); 2 Nanjing Anji Biotechnology Co. Ltd., Nanjing 210046, China

**Keywords:** integrin, αvβ3, α5β1, rheumatoid arthritis, angiogenesis, antagonist

## Abstract

ανβ3 and α5β1 are essential glycoproteins involved in the pathogenesis of rheumatoid arthritis (RA). Understanding of the role these integrins play in disease have been analyzed via description of cells-expressing ανβ3 and α5β1 and their mediators to trigger inflammation. ανβ3 and α5β1 facilitate cells-ECM and cell-cell communication, producing pro-inflammatory factors. Pro-inflammatory factors are essential for the building of undesirable new blood vessels termed angiogenesis which can further lead to destruction of bones and joints. Despite many attempts to target these glycoproteins, there are still some problems, therefore, there is still interest in understanding the synergistic role these integrins play in the pathogenesis of RA. The purpose of this review is to gain insights into the biological effects of ανβ3 and α5β1 in synovial tissues that are relevant to pathogenesis and therapy of RA.

## 1. Introduction

Rheumatoid arthritis (RA) is a chronic autoimmune disease with joints inflammation associated with synovitis, pannus formation and cartilage damage [1]. It involves extreme progressive bone resorption that often ultimately results in articular bone erosion and periarticular bone demineralization [2]. These conditions can impair other non-joint body systems such as chest, nerves, skin and eyes [3]. Moreover, it can also affect blood vessels and other important organs, including the liver and spleen [4]. Synoviocytes and infiltrated immune cells mediate immune response disorders in RA [5]. Nevertheless, the mechanistic basis of RA pathogenesis has not been fully elucidated. Insight into the molecular pathogenic mechanisms must still be understood. Integrins occur within RA pathogenesis and facilitate extracellular protein communication and inflammation of synovial cells, resulting in pathological intracellular signaling mediators. Additionally, integrins encourage cellular feedback through inflammation, osteoporosis, angiogenesis and apoptosis resistance by regulating cell proliferation and migration [6,7].

**Figure 1:**
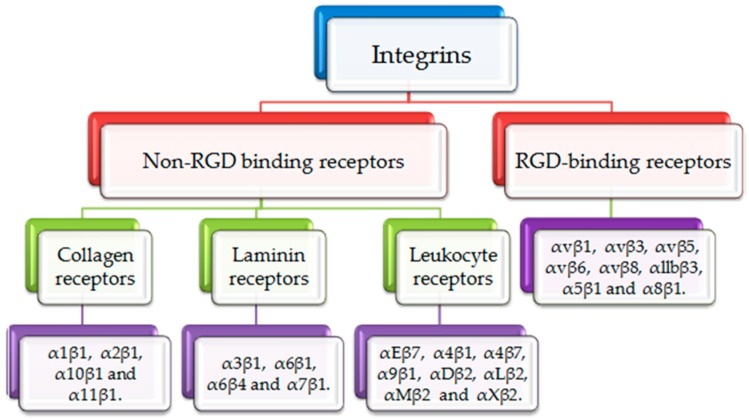
Classification of integrins. Integrins are divided into RGD binding receptors and non-RGD binding receptors. RGD binding receptors include αvβ1, αvβ3, αvβ5, αvβ6, αvβ8, αllbβ3, α5β1 and α8β1. Non-RGD binding receptors include collagen binding receptors (α1β1, α2β1, α10β1 and α11β1), laminin binding receptors (α3β1, α6β1, α6β4 and α7β1) and leukocyte binding receptors (αEβ7, α4β1, α4β7, α9β1, αDβ2, αLβ2, αMβ2 and αXβ2).

Integrins are heterodimeric adhesion glycoproteins that serve as signaling receptors. These heterodimeric structures consist of two different subunits, an α subunit and a β subunit. α/β subunits have particular extracellular matrix (ECM) protein binding sites to regulate essential cellular survival, motility, migration, inflamed responses and invasion [8]. There are eighteen α subunits and eight β subunits. These subunits together form twenty-four integrin molecules [9]. Based on the variety of ligands, integrins often can be classified into two groups as illustrated in Figure 1. Arg-Gly-Asp (RGD) binding receptors and non-RGD binding receptors. RGD receptors comprise α5β1, α8β1, αvβ1, αvβ3, αvβ5, αvβ6, αvβ8, and αllbβ3. Non-RGD receptors are subdivided into three categories; non-RGD binding collagen receptors: α1β1, α2β1, α10β1 and α11β1, non-RGD laminin receptors: α3β1, α6β1, α6β4 and α7β1 and non-RGD leukocyte receptors: αEβ7, α4β1, α4β7, α9β1, αDβ2, αLβ2, αMβ2 and αXβ2 [10,11].

Integrins participate in the immune response against infection and autoimmune diseases. Many integrins are expressed in monocytes, neutrophils, T cells, B cells, natural killer (NK) cells, macrophages, dendritic cells and platelets. The roles of αvβ3 and α5β1 in immunity are revealed by their contribution to immune cell migration and cell-cell interactions to induce an efficient immune response. Accumulation of evidence from human and mouse models experiments have been confirmed and indicated that defects in αvβ3 and α5β1 integrin expression or activation in the immune cells result in serious immunodeficiency or autoimmune diseases [12,13].

Despite the fact that the majority of integrins have been implicated in the pathophysiology of RA, we will only focus on fibronectin receptors, αvβ3 and α5β1. The functional homology of αvβ3 and α5β1 have been reported through their coordination and cooperation. The presence of both integrins plays an integral part in regulating myosin II activation in substrate rigidity sensing and cellular migration signaling. αvβ3 and α5β1 are un-separated supportive molecules of traction forces and actin cytoskeleton remodeling as a response to cyclic stretching and stiffening of ECM. The complete absence of α5β1 can be compensated by expression of αvβ3 [14]. Furthermore, αvβ3 and α5β1 represent a coordinated system for the function of each other. They regulate signal transduction of cell signaling cascade. Engagement of α5β1 causes calmodulin-dependent kinase II (CAMKII) activation that is a mediator of α5β1 cells migration, but ligation of αvβ3 inhibits CAMKII activation to block α5β1-influenced migration [15]. Moreover, α5β1 reinforces in vivo and in vitro endothelial cells migration during angiogenesis by αvβ3 [16].

Interestingly, αvβ3 and α5β1 are highly expressed in an inflammatory environment [17]. Upon inflammation, fibroblasts highly express αvβ3 or α5β1, thus is accompanied by increase pro-inflammatory mediators secretion such as matrix metalloproteinases (MMPs) and osteoclast activator, receptor activator of NF-κB ligand (RANKL) [18]. Moreover, fibroblast α5β1 ligation increases synthesis of B-lymphocyte activating factor (BAFF) [17]. BAFF interacts with BAFF-R and induces NF-κB signaling pathways. This interaction delivers signals for maintenance and survival of B cells [19]. ανβ3/lymphocytes or α5β1/lymphocytes adhesion to ECM ligands induces production of inflammatory factors that enhance survival and proliferation of synoviocytes and chondrocytes, causing synovial tissue hyperplasia and destruction of bone and cartilage [17]. αvβ3 have a direct effect on bones through its implication in osteoclastogenesis and bone resorption (bone loss). αvβ3 is over-expressed on osteoclasts and macrophages that are highly associated with bone destruction in RA joints [20]. Moreover, ανβ3 and α5β1 have a key role in RA angiogenesis regulation [21]. ανβ3 blockage with monoclonal antibodies (mAb) or small molecules reduces synovial tissue hyperplasia and causes RA regression [22,23,24]. This review will focus on the role of αvβ3 and α5β1 in the pathogenesis of RA and their pivotal physiological processes have attracted the interest of researchers. Additionally, it will highlight cells-expressing αvβ3 and α5β1, their mechanical stimulation of RA progression and briefly discuss the therapeutic antagonist strategies in targeting of this pair of integrins.

## 2. αvβ3 and α5β1 in RA Development

RA is described as a vicious disease characterized by joints inflammation and angiogenesis [25]. From this standpoint, the following sections present involvement of αvβ3 and α5β1 with facilitating ECM protein-rheumatoid cells communication during RA pathogenesis as well as angiogenesis-regulated factors.

### 2.1. αvβ3 and α5β1 Facilitate ECM Protein-Rheumatoid Cells and Cell-Cell Communication

**Figure 2:**
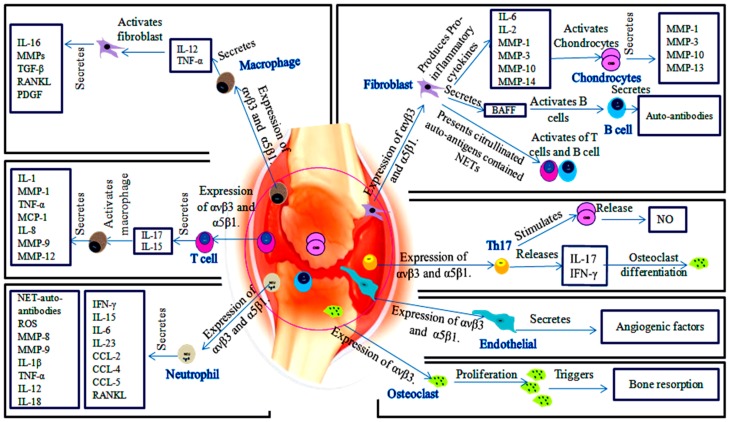
The role of αvβ3 and α5β1 in the development of RA. ECM proteins communication with cells-expressed αvβ3 and α5β1 as well as between αvβ3 and α5β1 of synovial cells, inducing bone and cartilage destruction. Fibroblasts-expressed αvβ3 and α5β1 secrete cytokines that induce MMPs production by chondrocytes, also fibroblasts activate B cells and T cells. αvβ3 and α5β1-expressed macrophages promote fibroblasts activation and secretion their cytokines. αvβ3 and α5β1-expressed T cells induce secretion of macrophages cytokines via IL-15 and IL-17. Neutrophils-expressed αvβ3 and α5β1 induce a group of pro-inflammatory cytokines, chemokines and MMPs. Osteoclasts express only αvβ3. αvβ3 enhances osteoclasts proliferation and bone resorption.

Roles of αvβ3 and α5β1 has been described in RA synovial tissue as illustrated in Figure 2. αvβ3 and α5β1 are expressed on the surface of synoviocytes (fibroblasts, endothelial cells and chondrocytes) and synovial-infiltrated cells (T cells, B cells, macrophages and neutrophils) [17,26,27]. αvβ3 and α5β1-expressed fibroblasts are active drivers of joints and cartilage destruction. Actually, the increase in number of fibroblasts is accompanied by excess cytokines secretion, including IL-6, IL-8, MMP-1 and MMP-3 [7]. These integrins facilitate invasion and attachment of fibroblasts to cartilage-pannus junction and induce production of MMPs and cathepsins. Invader fibroblasts drive chondrocytes to secrete MMPs [7,28]. Particularly, MMP-1, MMP-3 and MMP-10 are produced by fibroblasts and chondrocytes, MMP-14 is produced by fibroblasts, whereas MMP-13 is produced by chondrocytes [29,30]. α5β1-expressed fibroblasts indirectly induce B cells proliferation by increasing BAFF synthesis [17]. Additionally, fibroblasts are able to present citrullinated auto-antigens-contained neutrophils extracellular traps (NETs) to activate B cells and T cells [31].

Furthermore, fibronectin serves as αvβ3 and α5β1 ligand and up-regulated in inflamed articular tissues [17]. Loeser and Forsyth et al. reported that injection of fibronectin fragments (FN-f) into rabbit joints displayed its interaction to α5β1 on chondrocytes. α5β1/FN-f interaction induces cartilage damage and proteoglycan destruction by stimulating secretion of MMP-2, membrane type-1 matrix metalloproteinase (MT1-MMP) or MMP-3 [28,32]. On the other hand, Itoh and his colleagues confirmed that MT1-MMP over-expressed in inflamed synovial milieu at the pannus-cartilage junction and neutralizing DX2400 antibody to MT1-MMP inhibited the development of cartilage erosion in collagen-induced arthritis (CIA) mice. Therefore, MT1-MMP is an important factor in RA progression [33]. α5β1/chondrocytes response to FN-f leads to activate signaling proteins such as proline-rich tyrosine kinase 2 (Pyk2), Rac1 and mitogen-activated protein kinase (MAPK). These signaling proteins result in the production of nitric acid (NO), prostaglandin E (PGE) and vascular endothelial growth factor (VEGF) as well as increase the production of MMP-3 and MMP-13. These factors result in expressing of chondrocytes Toll-like receptor (TLR) [28,30].

Attracted macrophages are involved in activation of inflammatory synovial cells, secretion of matrix-degrading enzymes and neovascularization. Macrophages and T helper (Th) cells-expressing αvβ3 and α5β1 produce IL-17, IL-1 and TNF-α cytokines. These cytokines led to synovial fibroblasts activation to secrete pro-inflammatory cytokines such as MMPs, IL-6, tumor growth factor-β (TGF-β), RANKL and platelet-derived growth factor (PDGF) [1,7,34]. On the other hand, macrophages produce IL-1, TNF-α, IL-8, macrophage inflammatory protein 1 (MIP-1) and monocyte chemoattractant protein 1 (MCP-1) induced by IL-17 and IL-15 of T cells [35]. Additionally, macrophages produce MMP-9 and MMP-12 that collaborate with cytokines to enhance inflammatory cells migration and induce angiogenesis [36].

Notably, αvβ3 acts to increase monocytes adhesion to ECM and augment MMP-1, MMP-7 and MMP-10 secretion [37]. Then, it facilitates endothelial migration on intracellular adhesion molecule-1 (ICAM-1). In contrast, inhibition of αvβ3 on macrophages reduces endothelial adhesion via platelet endothelial cell adhesion molecule (PECAM-1) [38].

Granulocyte-macrophage colony-stimulating factor (GM-CSF) is produced by macrophage, neutrophil and Th17. RANKL and GM-CSF play an essential role in the control of osteoclasts differentiation, which express high levels of αvβ3. αvβ3 plays an important role in bone resorption as a result of osteoclasts migration by recruiting c-Src kinase, which phosphorylates p130, Pyk2 and paxillin [39]. Blocking of RANKL in rats adjuvant arthritis showed inhibition of bone and cartilage destruction [40].

Moreover, neutrophil is able to express αvβ3 and α5β1, which assist neutrophil migration [27]. Thereafter, neutrophils help in the progress of inflammation through the release of pro-inflammatory cytokines, reactive oxygen species (ROS) and NETs. NETs potentially affect on neutrophils and other inflamed cell types [41]. α5β1 and ανβ3 mediate cell adhesion to NETs [27]. NETs serve as a major stimulator of auto-antibodies production against citrullinated auto-antigens which trigger the development of RA. In addition, interaction of neutrophils with other cells induces production of MMP-8 and MMP-9, cytokines (IL-1β, TNF-α, IL-12, IL-18, IL-15, IFN-γ, IL-6, GM-CSF and IL-23), chemokines (CCL-2, CCL-4 and CCL-5) and RANKL [27,31]. MMPs extend the lifespan of neutrophils, this extension promotes synoviocytes migration and invasion, activates RANKL/RANK binding and initiates the angiogenic responses [42].

Th17 cells expressed αvβ3, which enables Th17 attachment to osteopontin (OPN). OPN serves as co-stimulator of IL-17 [34]. IL-17 indirectly enhances osteoclasts generation from macrophage lineages and induces production of NO in chondrocytes [35]. Interactions of Th17 cells with synovial cells express a repertoire of pro-inflammatory cytokines such as IL-17, IL-22, IL-26, IFN-γ, TNF-α, GM-CSF, CCL20, RANKL and MMPs [34,43]. Therefore, Th17 contributes in degradation of cartilage and bone. Neutralization of αvβ3 prevents osteoclasts-mediated bone destruction by attenuating Th17 activation and RANKL levels [34]. Furthermore, α5β1/fibronectin binding promotes proliferation of naïve T cells and memory T cells [17].

Communication of endothelial cells with ECM plays an important role in joints inflammation as well as endothelial cells represent the main angiogenic cells. The angiogenesis section highlights the roles of endothelial cells and their cytokines in maintaining the dysregulated integrins response that leads to RA.

Interaction of integrins with urokinase plasminogen activator receptor (uPAR) activates Rho GTPase to promote cell migration and invasion. uPAR/uPA binding converts plasminogen to plasmin that, in turn, degrades ECM components and activates MMPs [44]. αvβ3 and α5β1 regulate MMPs expression as the following, αvβ3 α subunit coupled to Fyn and Yes. Fyn and Yes activated FAK, which is a necessary element in SHC activation. SHC combined with Ras/Erk/MPAK that are activated from αvβ3/RTK receptors combination, thus activate MMPs [12]. By v/Src-transformed fibroblast, α5β1 up-regulated MMP-9 and MMP-2 through FAK-JNK pathway. Furthermore, αvβ3 and α5β1-stimulated cytokines bind to their receptors, causing MAPK and JAK/Stat pathway activation to regulate MMPs expression [42].

GPCR regulate the ECM proteins expression by G12/G13 and RhoA, supporting the engagement of ECM proteins with integrins. The regulation of FAk and MAPK activity by integrins and Gq/11 and G12/13, respectively in fibroblasts and endothelial cells leads to activation of PI3K/AKt and PKC pathways [45,46]. In addition, β1 and β3 integrins co-localize with the µ-opioid receptor in the cells and control receptor signaling, certainly by changing its pairing to either Gαs or Gαi proteins [47]. Moreover, binding of chemokines to GPCR on neutrophils induces activation of intracellular signaling pathways that activates integrins almost immediately. The activation of integrins downstream of GPCR engagement is referred to as inside-out signaling [48].

### 2.2. Angiogenesis

Angiogenesis plays an important role in persistence and pathology of RA. Angiogenesis is the generation of new venules or capillaries from pre-existing blood vessels. It is essential in wound healing, embryonic development; however, it is also associated with tumor metastasis and inflammatory diseases, including RA. Angiogenesis primarily depends on the interactions between endothelial cells, growth factors and ECM proteins [49,50]. The arthritic medium involves a large number of inflammatory cells and angiogenic effector molecules. VEGF and angiopoietins are main types of angiogenic factors that regulate angiogenesis process [25]. During RA inflammation, the synovial tissue expands, therefore, the supply of blood becomes inadequate, resulting in hypoxia. The excess of hypoxic state imposes activation of hypoxia-inducible factor-1 (HIF-1), leading to release of VEGF and then inducing the creation of new blood vessels to prevent arthritic hypoxia [51]. These conditions are accompanied by attraction and proliferation of fibroblasts, neutrophils and macrophages. The attracted cells release NO and a group of cytokines such as macrophage migration inhibitory factor (MIF), IL-18, IL-1, TNF-α, IL-6, IL-17, granulocyte-colony stimulating factor (G-CSF), GM-CSF and oncostatin M, leading to stimulation of VEGF, fibroblast growth factor-2 (FGF-2), hepatocyte growth factor (HGF) and TGF-β [50,52,53,54]. TGF-β and anaplastic lymphoma kinase-1 (ALK-1) interact with α5β1, promoting endothelial migration, survival and vessels formation via smad5/8 signaling [55]. α5β1 and αvβ3 are expressed in response to FGF-2 and VEGF on the surface of endothelial cells [21]. VEGF is remarkably higher in synovial fluids and serum of RA patients than patients with osteoarthritis (OA) [56].

Cytokine inhibitors such as TNF-α and IL-6 act on control of synoviocytes and synovial-infiltrated cells activation and are considered as effective agents in suppressing RA inflammation [57]. αvβ3 and α5β1 integrins promote endothelial cells migration and survival during an invasion of inflamed tissue, resulting in the creation of new vessels sprouts [54]. αvβ3 activates MMP-2, MMP-9 and urokinase plasminogen activator (uPA) production which induce ECM destruction [21,51]. Simic D et al. reported that MMP-2 collaborates with MMP-9 to induce CD40L. CD40L binds to α5β1 on platelets and activates platelets angiogenic effects [58]. Upon activation, platelets play in joints inflammation through release their pro-inflammatory microparticles, which react with leucocytes. In addition, activated platelets secrete IL-1 [59,60]. Evidence from RA patients pointed out that rheumatoid platelets produce higher amount of CD40L and P-selectin. Both correlated with anti-citrullinated protein antibodies [61]. In addition, α5β1 recruits mesenchymal stem cells migration by phosphorylation of platelet-derived growth factor receptors (PDGFR-β), which regulates PI3K-Akt [62]. α5β1-directed adhesion promotes αvβ3-mediated endothelial cell migration and survival in vivo and in vitro by inhibiting protein kinase A (PKA) activity [54].

**Figure 3:**
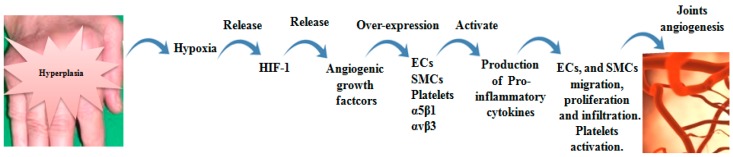
Role of αvβ3 and α5β1 in RA joint angiogenesis. Joint hyperplasia resulted from accumulated of synovial cells and their secretions, leading to extensive hypoxia. Hypoxia conditions lead to HIF-1 release, which is recognized as a stimulator of angiogenic growth factors (VEGF, FGF-2 and PDGF). The growth factors induce αvβ3 and α5β1 over-expression on endothelial cells (ECs), smooth muscle cells (SMCs) and platelets. Up-regulated αvβ3 and α5β1 activate production of pro-inflammatory cytokines that mediate ECs and SMCs migration and proliferation and platelets activation. These events entrain new vascularization.

Accumulated evidences indicated that α5β1-null endothelial cells demonstrate reduced proliferation, decreased vascularization and increased apoptosis. By small GTPase Rap1, VEGF-A/αvβ3 interaction, likewise, VEGFR-2/αvβ3 complex activates endothelial cells proliferation, migration and cell survival [63,64]. Gao et al. proved that αvβ3 is more effective in TNF-α-treated endothelial cells migration than α5β1. αvβ3 is adequately expressed on activated endothelial cells compared with α5β1 on resting endothelial cells [64]. However, Avraamides et al. revealed that α5β1 is insufficiently expressed on resting endothelial cells, but its expression is significantly increased on activated humans and mice endothelial cells [54]. PDGF-BB, VEGF and FGF-2 augment smooth muscle cells α5β1 expression. Moreover, FGF increases αvβ3 expression in gathered vessels. α5β1 and αvβ3 are regulator effectors for proliferation and migration of smooth muscle cells via up-regulated activation of focal adhesion kinase (FAK) [65]. *In vivo*, inhibition of VEGF reduced joint destruction through preventing endothelial cells migration, differentiation and tube formation [66]. Likewise, αvβ3 and α5β1 antagonists recover inflammation by inducing apoptosis of undesirable sprouting blood vessels [25]. The role of αvβ3 and α5β1 in RA joint angiogenesis was summarized in Figure 3.

## 3. Targeting of αvβ3 and α5β1 Integrins as Crucial Rheumatoid Arthritis Therapies

Targeting of αvβ3 and α5β1 integrins with antagonists could be a prospective field in RA treatment. In order to understanding αvβ3 and α5β1 selective antagonistic mechanisms, it is necessary to highlight the differences between αvβ3 and α5β1. α5β1 induces cells migration by Rho signaling, whereas αvβ3 induces cells migration by Rac [67]. Protein kinase Cα (PKCα) or Protein kinase Cε (PKCε) controls migration of cells-expressing α5β1 in complex with receptor for activated C kinase 1 (RACK1). In contrast, Protein kinase Cβ (PKCβ) is the regulating protein for αvβ3-expressing cells migration [15]. The inhibition of αvβ3 and α5β1 with antibodies, peptides, peptidomimetics or small molecules may become an effective alternative manner in the pharmacologic intervention of drugs for rheumatoid arthritis instead of conventional drugs including disease-modifying anti-rheumatic drugs, glucocorticoids and non-steroidal anti-inflammatory drugs are accompanied by deficiencies such as lose of effectiveness over time and serious side effects. However, only one of these integrins antagonists has been approved, Abciximab (anti-αvβ3 and anti-αllbβ3). Others still under evaluation as presented in Table 1. αvβ3 and α5β1 integrins represent safe targets because their over-expression is pertinent topathological angio- genesis and tumor cells [68,69].

Administration of αvβ3 antagonists to antigen-induced arthritic models inhibited synovial tissue angiogenesis, infiltration of inflammatory cells and destruction of cartilage and bone [20]. In fact, αvβ3 and α5β1 receptors have attracted much interest in the searches for new anti-angiogenic agents, subsequently selective αvβ3 and α5β1 antagonists offered new therapeutic opportunities for treatment of various human diseases like tumors, RA and osteoporosis [70]. Although, some of αvβ3 and α5β1 therapeutic antagonists exhibited good bioavailability in clinical trials, there are still some challenges that prevent approval of these antagonists. Extent target validation for RGD integrins can complicate the targeting process of αvβ3 and α5β1. αvβ3 and α5β1 have been shown to be over-expressed in many human diseases such as RA, cancers, fibrosis, ophthalmic states as well as being linked to disease development. The target validation for these two targets in RA is so expansive, with the contributions of αvβ3 and α5β1 not only in inflammatory angiogenesis but also in progress of bone resorption and synovitis [71]. Researchers focus primarily on the activity of the antagonist, without giving the physicochemical properties, permeability and selectivity more consideration. For example, SB-273005, anti-αvβ3 inhibitor showed low permeability and high toxicity. In addition, αvβ3 and α5β1 are expressed from the same cells and both bind to fibronectin, making defining affinity and selectivity very complex [71]. Most αvβ3 and α5β1 antagonists have entered clinical trials for cancer therapy, but etaracizumab is the only antagonist which has entered phase II clinical trials in the treatment of RA patients, where it failed however to show good clinical benefits. Since, the pathophysiological mechanism of αvβ3 and α5β1 in RA is similar to that seen in cancer, especially in regards to the occurrence of inflammation and angiogenesis, it is expected that αvβ3 and α5β1 antagonists could be feasible and practical tools to treat RA [72].

### 3.1. Anti-αvβ3 Agents

Etaracizumab is a humanized anti-αvβ3 mAb which was engineered to maintain antibody-dependent ligand specificity. Preclinical studies showed that Etaracizumab efficiently inhibited αvβ3-mediated cellular migration, adhesion and proliferation. A phase I clinical trial revealed that this antagonist is selective for αvβ3has anti-angiogenic features through inhibition of TNF-α and FGF-2 as well as inducing new blood vessel apoptosis [22,73]. Etaracizumab entered phase II clinical trials as a medication of RA. It was demonstrated to diminish synovial angiogenesis and pannus formation in animal models. However, the phase II trial for human RA treatment has been halted. This might be due to the serious observed sside effect such as myocardial infarction, atrial fibrillation and thromboembolic event and limited efficacy of antiangiogenic factors in controlling disease progression [72,74,75]. Combination therapy using etaracizumab with anti-angiogenic factors or with anti-inflammation mediators like anti-cytokines may be a solution to overcome these negative outcomes.

Intetumumab (CNTO95) and DI17E6 are recognized as pan αv mAb, which targets all αv subunit/ligands connection including αvβ3. In preclinical evaluation, CNTO95 demonstrated in vivo and in vitro significant anti-tumor and anti-angiogenic activities. CNTO95 and DI17E6 hindered migration and adhesion of human umbilical vein endothelial cells (HUVECs) and human melanoma cells [51,76]. CNTO95 and DI17E6 exhibited a favorable safety profile in a phase I clinical trial, however, CNTO95 seems to be in a forefront before DI17E6 [77]. A randomized phase II study of CNTO95 in combination with docetaxel and prednisone for treatment of metastatic castration-resistant prostate cancer patients revealed shorter progression-free survival (PFS) without toxicity among castration-resistant prostate cancer patients [78].

Abciximab (c7E3) is a chimeric mAb antagonist of αvβ3 and αllbβ3. It is characterized by having anti-angiogenic and anti-tumor activity and has been approved by the FDA [79]. Cilengitide (EMD 121974) is a cyclic αv RGD pentapeptide which selectively blocks interactions between αvβ3 and αvβ5/ligands and αvβ3-mediated cell-cell binding. In a preclinical in vivo study, cilengitide attenuated the proliferation and migration of angiogenic endothelial cells and tumor cells in many solid tumors through inhibition of FAK-Src-Akt and Erk pathways, VEGF and NF-kB [51,76]. Phase I and phase II trials established encouraging safety and tolerability profiles for either cilengitide used as a single agent or in combination with radiation or chemotherapy [70,80]. Nevertheless, cilengitide failed in phase III clinical trials [71,81]. L000845704 (MK-0429), is the first small molecule αvβ3 inhibitor. Preclinical and phase I studies revealed favorable safety results for its ability to inhibit bone resorption [82]. SB273005 is a small molecule αvβ3 antagonist, which prevented in vitro endothelial cell migration and in vivo bone loss in an arthritic rat model in preclinical studies [51,76]. SB273005 inhibited bone and cartilage destruction in adjuvant-induced arthritis (AIA) rats [23]. Unfortunately, SB273005 failed in the treatment of osteoporosis when it entered the phase I stage [71]. SCH221153 is a RGD-based peptidomimetic αvβ3 and αvβ5 inhibitor. It has a high affinity to target αvβ3. SCH221153 inhibits adhesion of αvβ3 to ECM proteins and endothelial cells and to FGF-2 [83]. GLPG-0187 is a pan αv and α5β1 small molecule inhibitor which possesses anti-angiogenic, anti-tumor and anti-bone resorption effects in preclinical trials. However, GLPG-0187 as failed in phase I as an anti-cancer agent [84,85,86].

### 3.2. Anti α5β1 Agents

Volociximab is the first α5β1 inhibitor. It is a chimeric mAb which shows a high affinity for α5β1 [51]. It has the ability to inhibit α5/fibronectin interaction. During preclinical studies, volociximab induced in vitro and in vivo endothelial cell apoptosis and prevented blood vessel formation [87]. Anti-angiogenic and anti-tumor activities were revealed in chick chorioallantoic membrane (CAM) following volociximab inoculation. Volociximab/anti-VEGF combination lacked the anti-proliferative effect of volociximab for endothelial cells and therefore this antagonist acts independently without blocking growth factors. Phase I trials showed volociximab was well-tolerated and safe in humans. Through phase II trials, volociximab showed a similar tolerability, safety model and promising potential in treating cancer [88]. Further phase II and III clinical trials are needed to treat solid tumors resistant to available therapy [51]. PF-04605412 is a fully human mAb for α5β1 induced antibody-dependent cellular cytotoxicity (ADCC) which acts against endothelial cells and shows in vivo anti-angiogenic and anti-tumor effects in preclinical studies. PF-04605412 clinical development trials are were discontinued [89].

JSM6427 is a non-peptide α5β1 inhibitor. In preclinical evaluation, JSM6427 induced anti-proliferative activity for endothelial cells and prevented choroidal neovascularization. A phase I trial has reported that JSM6427 showed enhanced safety and tolerability profiles [24,51,90]. ATN-161 is a non–RGD peptide antagonist that blocks not only α5β1, but also αvβ3, significantly blunts macrophage activation, inhibits vascular cell adhesion protein 1 (VCAM-1) expression in atherosclerotic mice and reduces breast cancer metastasis [91,92]. In phase I trials, ATN-161 showed a good safety and tolerability profile. ATN-161 in combination with radiation and chemotherapy phase II data are not available yet [70,71]. Studies with α5β1 and αvβ3 specific antagonists demonstrated that simultaneous targeting of this dual integrin inhibited migration of smooth muscle cells and invasive proliferation. Similarly, combined blockade of α5β1 and αvβ3 as compared to αvβ3 alone induced apoptosis of endothelial cells and attenuated MMPs-dependent angiogenesis [16]. HM-3 is an inhibitor of α5β1 and αvβ3. It is shows anti-angiogenic activity through inhibition of inflammatory factors, VEGF and PDGF-A in endothelial cells. Phase I clinical trials are currently underway [25].

## 4. Conclusions and Future Perspectives

In this review, we have explained the role of αvβ3 and α5β1 integrins in RA. αvβ3 and α5β1 share similar binding ligand, structure, production sources and functional effects. These similarities assist αvβ3 and α5β1 to act as inflammatory and angiogenic factors in RA progression. αvβ3 and α5β1 are recognized as fibronectin receptors and in addition αvβ3 can bind to vitronectin, fibronegin, osteopontin and bone sialoprotein [6].

Moreover, structurally the density map of un-ligated α5β1 is similar to the configuration of the αvβ3 crystal structure [96]. αvβ3 and α5β1 are over-expressed in all synoviocytes and infiltrated immune cells, with the exception of osteoclasts which have never been reported to express α5β1. Therefore, there is the need to understand the mechanisms that explain osteoclasts failure to express α5β1. αvβ3 and α5β1 are concurrent molecules in various normal and pathological cellular events such as modulating angiogenesis.

In addition, α2β1, αvβ5, αllbβ3 and α1β1 integrins are recognized as angiogenesis stimulators. It is being envisaged if the current therapy technologies (immunotherapy, genetic, radiation and chemotherapy) can target all these integrins simultaneously. In comparison, immunotherapy is safer than the others with lesser side effects. It is possible to use more than one of these technologies to block all or most of angiogenic integrins at the same time. αvβ3 and α5β1 are implicated in angiogenesis and inflammation of RA. Their participation in synovial cell proliferation, differentiation and migration enhances secretion of pro-inflammatory and angiogenic factors, making them appropriate therapeutic targets. Many αvβ3 and α5β1 inhibitors have been studied, evaluated and discussed. It is noteworthy that many αvβ3 antagonists actually target both αvβ3 and α5β1, and that dual αvβ3/αllbβ3 antagonists have been developed. Although, most of αvβ3 and α5β1 therapeutic antagonists elicit better bioavailability during clinical trials for cancer and other diseases, most of these inhibitors have been not assessed for RA despite the biologically similar effects of αvβ3 and α5β1 on cancer and RA. Thus, αvβ3 and α5β1 are still potential therapeutic targets for treatment RA and more research should be done in this regard.

## Figures and Tables

**Table 1:** Summary table of αvβ3 and α5β1 integrin antagonists.

Target	Antagonist Name	Antagonist Type	Effect on Cells Response (Functions)	Clinical Trials Phase	Ref.
**αvβ3**	Etaracizumab	Engineered mAb	Inhibited cellular migration, adhesion and proliferation. Induced blood vessels apoptosis. Anti-angiogenic activity via blocking FGF-2 and TNF-α.	Phase II for RA, solid tumors, lymphoma and psoriasis.	[71,73,77]
	Intetumumab (CNTO95)	mAb	Inhibited HUVECs migration and adhesion of melanoma cells.	Phase II for solid tumors.	[51,71,78]
	DI17E6	mAb	Inhibited HUVECs migration and adhesion of melanoma cells. Suppressed development of prostate cancer.	Phase I for solid tumors.	[70,71,93,94]
	Abciximab (c7E3)	Chimeric mAb	Inhibited platelet aggregation by binding to αvβ3 and αllbβ3. Anti-tumor activity.	Approved for cancer therapy.	[51,71,79]
	Cilengitide (EMD121974)	RGD-peptide	Attenuated endothelial cells and tumor cells proliferation and migration by inhibiting the FAK/Src/AKT and Erk pathway. Induced apoptosis in endothelial cells.	Failure in phase III for cancer.	[71,80,81]
	L000845704 (MK-0429)	Small molecule	Inhibited bone resorption.	Phase I for osteoporosis and prostate cancer.	[71,82]
	SB273005	Small molecule	Inhibited endothelial cells migration and bone loss.	Failure in phase I for osteoporosis.	[23,71]
	SCH221153	RGD-peptide mimetic	Inhibited endothelial cells disorders and FGF-2 inhibitor.	-	[71,83]
	GLPG-0187	Small molecule	Anti-angiogenic. Anti-tumor. Anti-bone resorption.	Phase I for solid tumors.	[70,71,85,86]
	HM-3	RGD-peptide	Inhibited inflammatory factors, VEGF and PDGF-A in endothelial cells.	Phase I for cancer.	[25]
**α5β1**	Volociximab	Chimeric mAb	Induced in vivo and in vitro endothelial apoptosis. Prevented blood vessels formation.	Phase II for cancer.	[51,88]
	PF-04605412	mAb	Exhibited anti-angiogenesis and anti-tumor properties.	Phase I for cancer.	[93,95]
	JSM6427	Small molecule	Induced anti-proliferative of endothelial cells activity. Showed an inhibition of choroidal neovascularization.	Phase I for age-related macular degeneration (AMD).	[90]
	ATN-161	Non-RGD peptide	Blunted macrophage activation. Inhibited CAM expression. Exhibited anti-angiogenic properties.	Phase II for renal cancer.	[70,92]
	HM-3	RGD-peptide	Inhibited inflammatory factors, VEGF and PDGF-A in endothelial cells.	Phase I for cancer.	[25]
